# Artificial intelligence models and combined scoring approaches for endometrial receptivity assessment in *in vitro* fertilization

**DOI:** 10.3389/frai.2025.1673800

**Published:** 2026-03-02

**Authors:** Teymur Bornaun

**Affiliations:** Department of Obstetrics and Gynecology, Istanbul Bagcilar Training and Research Hospital, Istanbul University Health Sciences, İstanbul, Türkiye

**Keywords:** *in vitro* fertilization (IVF), artificial intelligence (AI), endometrial receptivity, embryo implantation, deep learning models, predictive scoring systems, reproductive medicine, assisted reproductive technologies (ART)

## Abstract

This retrospective study evaluates the effectiveness of artificial intelligence (AI) models and integrated scoring systems in assessing endometrial receptivity during *in vitro* fertilization (IVF). We combine AI-driven image analysis for automated endometrial segmentation (U-Net with VGG16 encoder) and embryo quality classification (VGG16) with a patient-specific clinical probability metric (SART) to generate a composite score. Ultrasound images of the endometrium were processed using a standardized pipeline (denoising, normalization, ROI cropping, augmentation), enabling automated quantification of thickness, echogenicity/pattern, and derived measures. Embryo quality was assessed by a fine-tuned classifier benchmarked against expert embryologists. The composite score is defined as: CS = (EQS + ERS)/2 × PSART/10 where EQS is the AI-derived embryo quality score (0–10), ERS is the AI-derived endometrial receptivity score (0–10), and PSART is the SART pregnancy probability scaled to 0–10. In internal validation, the composite score demonstrated higher discriminative performance for biochemical pregnancy than individual components (AUC 0.94 vs. EQS 0.88 and ERS 0.85). While integrated scoring improved prediction relative to single-source models, generalizability is limited by the single-center, retrospective design and modest dataset size augmented synthetically. Prospective, multi-center validation with live-birth outcomes and incorporation of explainable AI (e.g., saliency/attribution maps) are needed to support clinical deployment. These findings suggest that AI-based models, when embedded in multidimensional scoring frameworks, may help optimize embryo transfer timing and support precision IVF, complementing—not replacing—clinical expertise.

## Introduction

Infertility remains a major public health challenge, affecting approximately one in every six couples of reproductive age worldwide, with global fertility rates continuing to decline as couples increasingly seek assisted reproductive technologies (ART) to conceive (Sharma et al., 2019; Wang et al., 2019). Among ART procedures, *in vitro* fertilization (IVF) is the most widely practiced and has become the cornerstone of treatment for patients experiencing difficulty achieving pregnancy. Despite significant advances—including personalized ovarian stimulation protocols, extended embryo culture, preimplantation genetic testing, and improvements in embryo selection—only about one-third of all initiated IVF cycles result in a clinical pregnancy (Barrera et al., 2023). A comprehensive understanding of infertility etiology, coupled with careful patient evaluation and a multidisciplinary clinical approach, is therefore essential for optimizing treatment outcomes (Barrera et al., 2023; Raimundo and Cabrita, 2021).

The IVF process involves multiple critical steps: controlled ovarian stimulation, oocyte retrieval, fertilization, embryo culture, and finally, embryo transfer. For successful implantation and pregnancy, an adequately prepared endometrium, a high-quality embryo, and well-timed embryo–endometrium communication must all be present (Raimundo and Cabrita, 2021; Rolfes et al., 2023). This precise temporal and molecular interplay is referred to as *endometrial receptivity*, a transient state during which the endometrium is most receptive to embryo implantation (Moghadam et al., 2022). The period of optimal receptivity, known as the “window of implantation” (WOI), occurs during a narrow time frame in the mid-luteal phase, and even minor deviations from this timing can result in implantation failure and reduced IVF success rates (Senapati et al., 2016; Tang et al., 2021; Fanton et al., 2023). It is now recognized that the timing of the WOI is not uniform among all women, and suboptimal receptivity may account for up to one-third of implantation failures (Mahutte et al., 2022; Yang et al., 2018).

Accurately identifying the optimal timing for embryo transfer has long been a key objective in reproductive medicine, but despite the introduction of advanced diagnostic tests and novel laboratory techniques, precise prediction of this critical window remains challenging (Parasnis and Bandaru, 2022). A range of clinical and laboratory parameters are currently used to evaluate endometrial receptivity, such as endometrial thickness and pattern assessed via transvaginal ultrasound (Ver Milyea et al., 2020), uterine blood flow parameters including pulsatility and resistance indices (Sapkota and Sharma, 2023), endometrial biopsy with histological dating (Craciunas et al., 2019), immunohistochemical expression of proteins such as integrins, leukemia inhibitory factor (LIF), and mucin-1 (MUC-1) (Rajpurkar et al., 2022), serum hormonal profiles (particularly progesterone and estradiol levels) (Calhaz-Jorge et al., 2016), and molecular markers including HOXA-10 and HOXA-11 (Murray et al., 2018) , which have been shown to be significantly altered in patients with PCOS, potentially impairing endometrial receptivity (Kara et al., 2019). Additional approaches, such as sonoelastography to assess endometrial stiffness (Choe and Shanks, 2016) and microbiome analysis (Lessey and Young, 2019), are emerging. No single method, however, can provide a definitive assessment of receptivity, which is why clinicians often rely on a combined evaluation of multiple complementary parameters (Ver Milyea et al., 2020; Sapkota and Sharma, 2023; Craciunas et al., 2019; Rajpurkar et al., 2022; Calhaz-Jorge et al., 2016; Murray et al., 2018; Choe and Shanks, 2016; Lessey and Young, 2019).

Traditional assessments are not only labor-intensive but also subject to significant inter-observer variability and lack reproducibility, particularly in ultrasound-based measurements such as endometrial thickness (Enciso et al., 2015; Zhang et al., 2022). These limitations have fueled the need for more standardized, automated, and objective evaluation methods. While conventional biomarkers tend to show high sensitivity, they often lack sufficient specificity, underscoring the need for alternative tools to predict non-receptive endometrial states (Heger et al., 2012).

In recent years, artificial intelligence (AI) and machine learning (ML) have emerged as promising technologies in reproductive medicine. Their applications include automated embryo grading, protocol optimization, and the prediction of implantation outcomes. AI models, trained on large and diverse datasets, offer the potential to enhance clinical decision-making, improve IVF success rates, and reduce time to pregnancy while alleviating both emotional and financial burdens for patients (Check and CDL, 1995).

The present study aims to develop an AI-based predictive framework for endometrial receptivity by integrating transvaginal ultrasound-derived parameters with embryo morphology and pregnancy probability scores. By combining traditional clinical markers with advanced computational analysis, we propose a composite scoring system designed to optimize embryo transfer timing, enhance implantation success, and advance the personalization of IVF treatment.

## Materials and methods

### Data sources and ethical considerations

This study was conducted with the collaboration of the University of Health Sciences Istanbul Bağcılar Training and Research Hospital and Bakırköy Dr. Sadi Konuk Training and Research Hospital. The research protocol was reviewed and approved by the Ethics Committee of Istanbul Bağcılar Training and Research Hospital (Approval No: 2023/482; Date: 15 March 2023) and the Ethics Committee of Bakırköy Dr. Sadi Konuk Training and Research Hospital (approval no: 2023/174; Date: 27 March 2023).

Since this investigation focused on algorithm development, all data were anonymized and retrospective in nature. No interventions or patient-identifiable data were used, and no direct impact on patient treatment decisions occurred (Paulson, 2019).

The dataset included 169 high-resolution transvaginal ultrasound (TVUS) images of the endometrium and 230 embryo microscopy images (including cleavage-stage and blastocyst images). All images were de-identified and standardized into TIFF/JPEG formats with a minimum resolution of 800 × 600 pixels.

Inclusion criteria for images were as follows:

- Clear visualization of the endometrial midline and uterine cavity in sagittal plane TVUS images.
- Embryo images obtained under light microscopy with morphological grading (e.g., Gardner classification).
- Image quality suitable for segmentation (contrast, focus, and absence of artifacts).

Exclusion criteria:

- Images with annotations, compression artifacts, or insufficient clarity.
- Embryo images without corresponding morphological scoring references.

As seen in Table 1, data augmentation tripled the available dataset size, which helped reduce overfitting and improve generalizability of the AI model (Schild et al., 2001). GAN-based synthetic image generation, particularly for embryo datasets, further diversified morphological patterns to mimic real-world variability (Schild et al., 2001; Alebić, 2022).

**Table 1:** Dataset composition and augmentation parameters.

Image type	Original images (*n*)	Augmented images (*n*)	Final dataset (*n*)	Augmentation techniques
TVUS (endometrium)	169	+507	676	Rotation ±15°, scaling, flipping, cropping
Embryo (cleavage stage)	120	+360	480	GAN-based synthesis, random shifts
Embryo (blastocyst stage)	110	+330	440	Brightness/contrast adjustments, flips
Total	399	+1,197	1,596	Combined augmentation methods

### Image preprocessing

All images underwent a standardized preprocessing workflow to ensure consistent quality before feeding into the AI pipeline. This workflow included noise reduction, pixel intensity normalization, and data augmentation to enhance feature clarity and improve the model’s robustness (Schild et al., 2001; Labarta et al., 2021).

**Figure 1:**
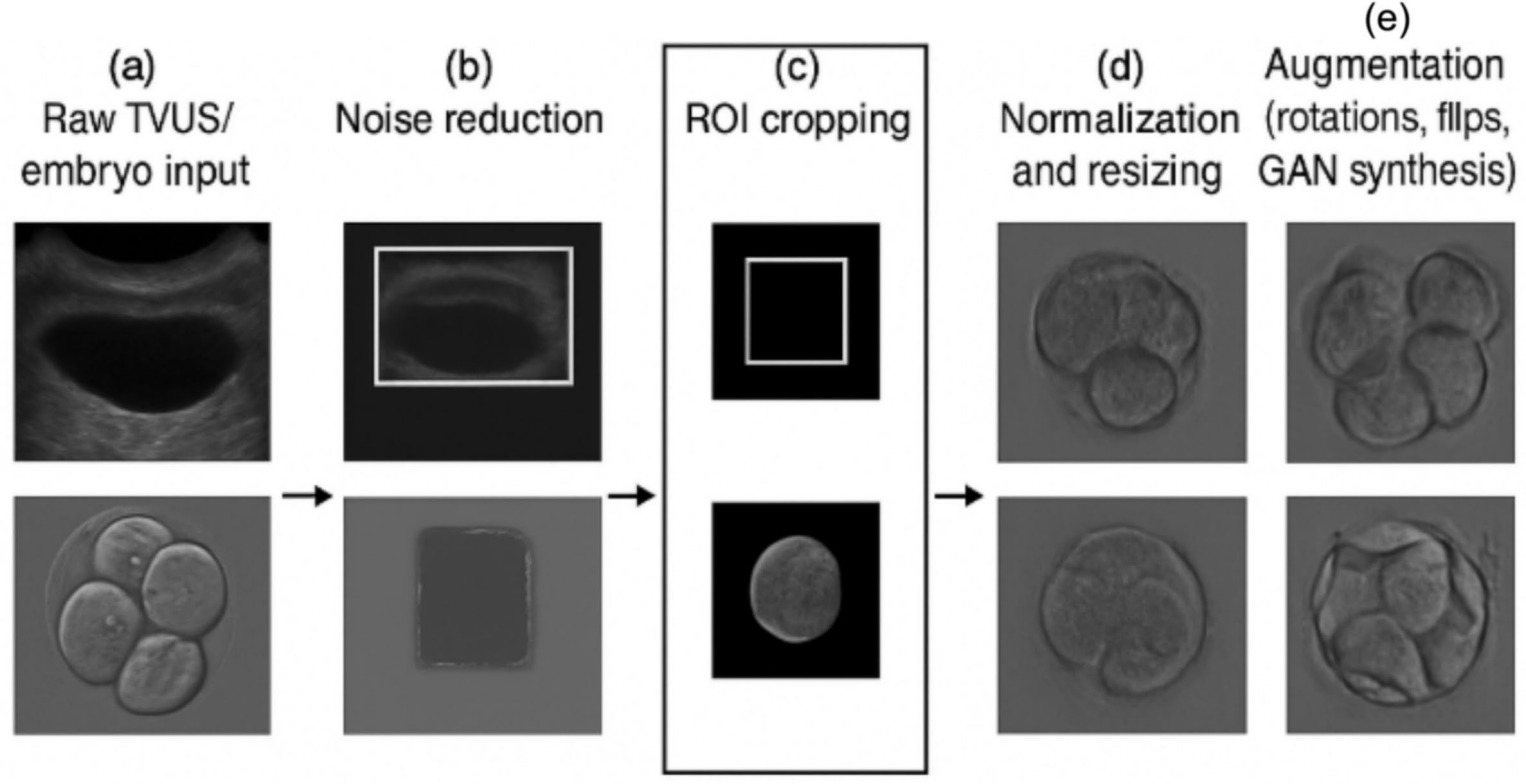
Image preprocessing pipeline for endometrial and embryo images. The figure depicts the five-step preprocessing workflow: **(a)** Raw TVUS/embryo input, **(b)** Noise reduction, **(c)** ROI cropping, **(d)** Normalization and resizing, **(e)** Augmentation (rotations, flips, GAN synthesis). This standardized pipeline improves image clarity and prepares data for segmentation and classification.

Preprocessing pipeline steps (Figure 1):

1. Noise reduction and normalization: TVUS and embryo images were first filtered using median and Gaussian filters to suppress speckle noise common in ultrasound imaging. Pixel intensities were normalized to a range of 0–255, enhancing the contrast between endometrial layers and surrounding myometrial tissue.
2. Cropping to region of interest (ROI): For TVUS images, cropping focused on the mid-sagittal uterine plane, ensuring the entire endometrial stripe from the fundus to the endocervical canal was captured. For embryo images, the ROI was centered on the blastocyst or cleavage-stage embryo with background suppression.
3. Resizing: images were resized to 224 × 224 pixels to align with the input dimensions of the VGG16 backbone used in the U-Net and classification networks (Alebić, 2022).
4. Augmentation: a combination of rotation (±15°), scaling, horizontal and vertical flips, random cropping, brightness/contrast shifts, and Generative Adversarial Networks (GAN)-based synthetic images was used. GANs significantly enhanced the diversity of embryo morphology in the dataset by producing realistic variations of blastomere patterns (Schild et al., 2001).
5. Annotation review: images containing artifacts, annotations, or improper focus were manually excluded.

Figure 1 illustrates the preprocessing pipeline for both TVUS and embryo datasets, highlighting each stage from raw image input to augmented image output.

The augmentation parameters were defined as follows: rotation between −20° and +20°, scaling factor 0.8–1.2×, horizontal flip (*p* = 0.5), random cropping between 85–100% of the image area, brightness adjustment ±15%, and contrast adjustment ±20%. Additionally, 1,000 paired embryo and endometrial images were used to train a CycleGAN for synthetic image generation to enrich morphological variability.

### AI model development

#### Endometrial segmentation

**Figure 2:**
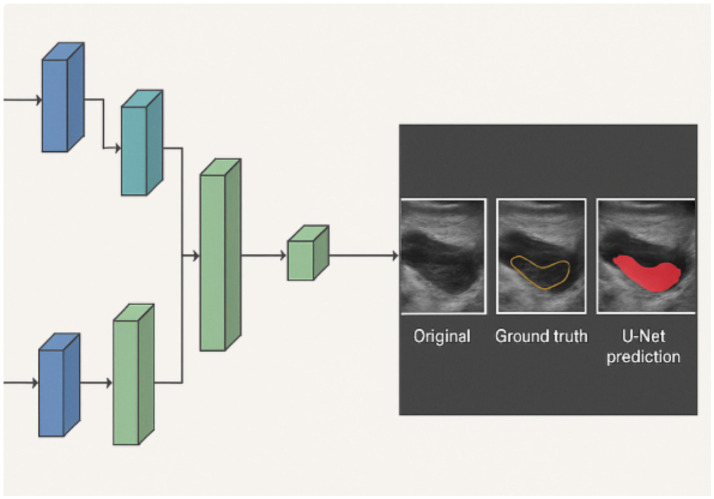
U-Net architecture and example segmentation output. The figure illustrates the encoder-decoder structure of the U-Net with skip connections. The right panel demonstrates an example of endometrial segmentation: original ultrasound image (left), ground truth annotation (middle), and U-Net prediction (right).

Endometrial segmentation was performed using a U-Net convolutional neural network (CNN) with a VGG16 encoder backbone, pre-trained on ImageNet to leverage transfer learning (Alebić, 2022). The U-Net model architecture comprised an encoder (contracting path) for feature extraction and a decoder (expanding path) for precise pixel-wise segmentation. Skip connections were used to preserve spatial information during upsampling (Figure 2).

Following segmentation, the model automatically measured:

- Endometrial thickness (mm): Maximum perpendicular distance from the basal layer at the midline.
- Endometrial pattern classification: Categorized into trilaminar, homogeneous, or hyperechoic based on texture and echogenicity.
- Endometrial volume (mL): Estimated from 2D images using 3D volume reconstruction algorithms.

##### Performance metrics

Segmentation performance was assessed using Dice similarity coefficient (DSC), Intersection-over-Union (IoU), sensitivity, and specificity. As shown in Table 2, the U-Net achieved a Dice score of 0.92 ± 0.02, indicating high accuracy in delineating endometrial boundaries.

**Table 2:** Endometrial segmentation metrics and parameter ranges.

Metric	Mean ± SD	Range
Dice Coefficient	0.92 ± 0.02	0.88–0.95
IoU	0.87 ± 0.03	0.82–0.91
Sensitivity (%)	91.4 ± 2.5	88.0–94.8
Specificity (%)	93.2 ± 2.1	90.1–96.0
Thickness (mm)	7.5 ± 1.2	5.2–10.8

#### Embryo quality assessment

Embryo images (cleavage-stage and blastocyst-stage) were analyzed using a fine-tuned VGG16 classifier. The model was trained to predict embryo quality scores (EQS) from 0 to 10 by assessing:

- Cleavage-stage embryos: blastomere symmetry, cytoplasmic fragmentation (%), zona pellucida integrity.
- Blastocysts: expansion stage (3–6), inner cell mass (ICM) quality, trophectoderm organization (Labarta et al., 2021).

AI-based predictions were benchmarked against expert embryologist scores, with agreement quantified using Cohen’s kappa (*κ* = 0.87), reflecting strong consistency.

#### Composite scoring framework

To integrate both uterine and embryonic parameters, we developed a Composite Score (CS) designed to predict implantation potential by combining:

Embryo quality score (EQS): determined by the AI-based VGG16 classifier (0–10 scale).
Endometrial receptivity score (ERS): derived from ultrasound-based parameters including thickness, pattern, and vascularity.
SART pregnancy probability score (PSART): a clinical probability score scaled to 10.

The final composite formula was expressed as:


$$\begin{array}{l} \mathrm{CS}=\mathrm{EQS}+\mathrm{ERS2}\times \text{PSART}1\mathrm{0CS}=\backslash \text{frac}\{\mathrm{EQS}+\mathrm{ERS}\}\{2\}\\ \backslash \text{times}\backslash \text{frac}\{\text{PSART}\}\{10\}\mathrm{CS}=\mathrm{2EQS}+\mathrm{ERS}\times 10\text{PSART} \end{array}$$

Interpretation:

- CS < 4: Low predicted implantation probability.
- CS 4–7: Moderate implantation probability, requiring clinical review.
- CS > 7: High implantation potential.

Table 3 outlines the detailed scoring components and weightings used to generate the composite score.

**Table 3:** Composite scoring framework integrating EQS, ERS, and PSART.

Parameter	Range	Score (0–10)	Weighting
Embryo quality (EQS)	0–10	AI-assigned	50%
Endometrial receptivity (ERS)	0–10	U-net derived	50%
SART probability (PSART)	0–10 (scaled)	Clinical input	Multiplier

As shown in Table 3, embryo and endometrial scores are averaged, and this value is scaled by PSART to yield the composite score.

#### Model training and validation

The AI models (U-Net and VGG16-based classifier) were trained and validated using 80% training data and 20% test data, ensuring balanced representation of all classes. Training was conducted on a workstation with NVIDIA RTX 3080 GPU and 64GB RAM.

Training details:

- Framework: PyTorch 2.0.
- Optimizer: adam (learning rate = 1 × 10^−4^). The Adam optimizer was configured with *β*₁ = 0.9, β₂ = 0.999, and a weight decay coefficient of 1 × 10^−5^. The learning rate decayed exponentially by a factor of 0.95 every 10 epochs. Hyperparameters were tuned via five-fold cross-validation and Bayesian optimization to minimize validation loss.
- Batch size: 32.
- Epochs: 100 with early stopping based on validation loss.
- Loss functions: dice loss for segmentation and cross-entropy for classification.
- Regularization: dropout layers (*p* = 0.5) and L2 weight decay were applied to avoid overfitting.

##### Performance evaluation

**Figure 3:**
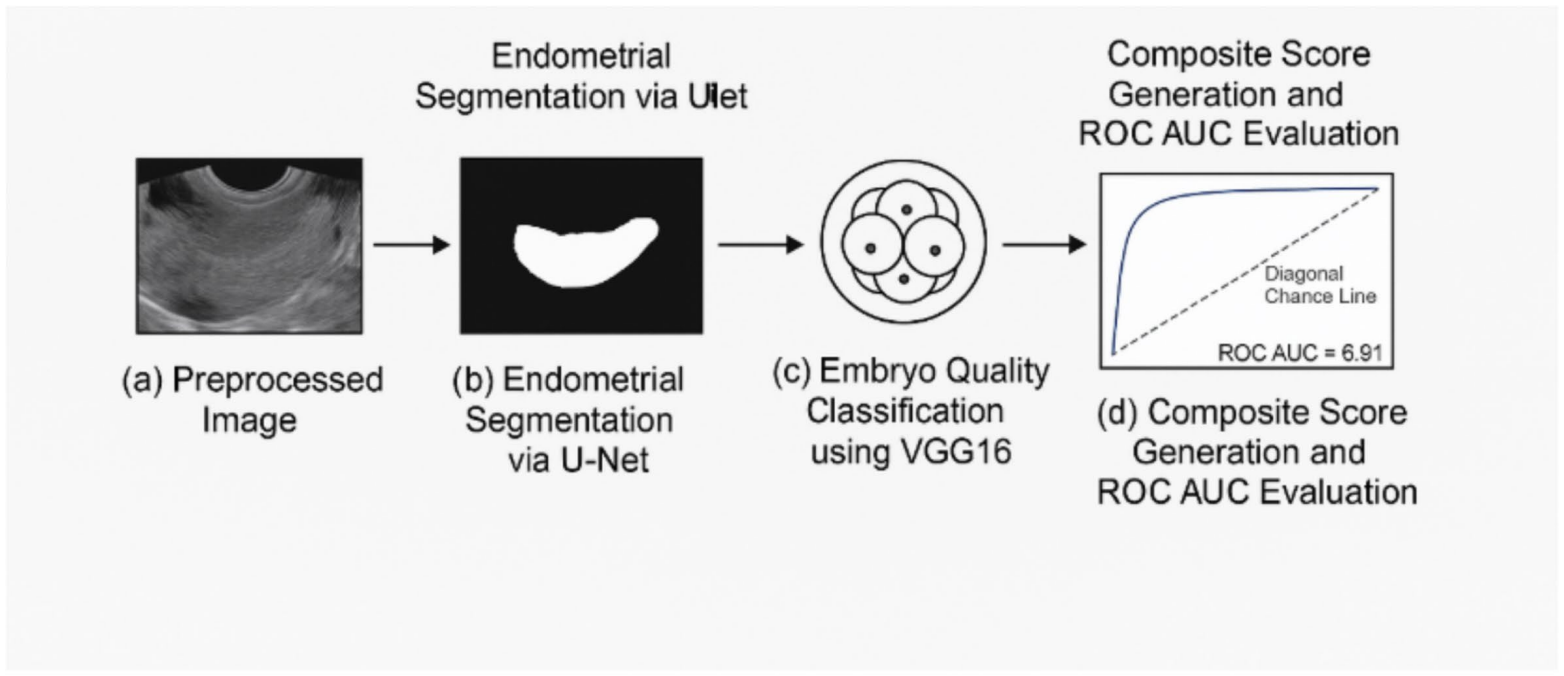
Model training and validation workflow. This figure demonstrates the sequential steps of the AI pipeline: **(a)** Input of preprocessed images, **(b)** segmentation via U-Net for endometrial measurements, **(c)** embryo embryo quality scores (EQS) ranged from 0–10 and were categorized as follows: classification using VGG16, and **(d)** final composite score generation and ROC-AUC evaluation.

Model performance was assessed using accuracy, sensitivity, specificity, F1-score, and the area under the ROC curve (ROC-AUC). Figure 3 illustrates the training workflow, including data preprocessing, U-Net segmentation, embryo quality classification, and composite score generation.

- High quality (EQS ≥ 8): symmetrical blastomeres, minimal fragmentation (< 5%), uniform cytoplasm, and intact zona pellucida.
- Medium quality (EQS 5–7): moderate expansion, minor fragmentation (5–15%), slightly uneven cytoplasm.
- Low quality (EQS < 5): irregular blastomeres, fragmentation > 20%, granular or vacuolated cytoplasm.

These thresholds are consistent with the gardner blastocyst grading system.

### Statistical analysis

All statistical analyses were performed using SPSS v24.0 (IBM Corp., Armonk, NY, USA) and Python (pandas, scikit-learn, and NumPy). Continuous variables were reported as mean ± standard deviation (SD), and categorical variables as frequencies and percentages. Group comparisons between receptive and non-receptive cases were conducted using Student’s t-test (for normally distributed data) or Mann–Whitney U test (for non-parametric data), while categorical variables were compared using the Chi-square test.

To assess the predictive performance of the composite score (CS), logistic regression models were constructed, and odds ratios (OR) with 95% confidence intervals (CI) were calculated. Model accuracy was further validated using Receiver Operating Characteristic (ROC) curve analysis, evaluating the area under the curve (AUC) as an indicator of diagnostic performance (Stanziano et al., 2023; Crha et al., 2019).

The performance metrics calculated included:

- Sensitivity (true positive rate)
- Specificity (true negative rate)
- Positive predictive value (PPV)
- Negative predictive value (NPV)
- F1-score and accuracy

**Figure 4:**
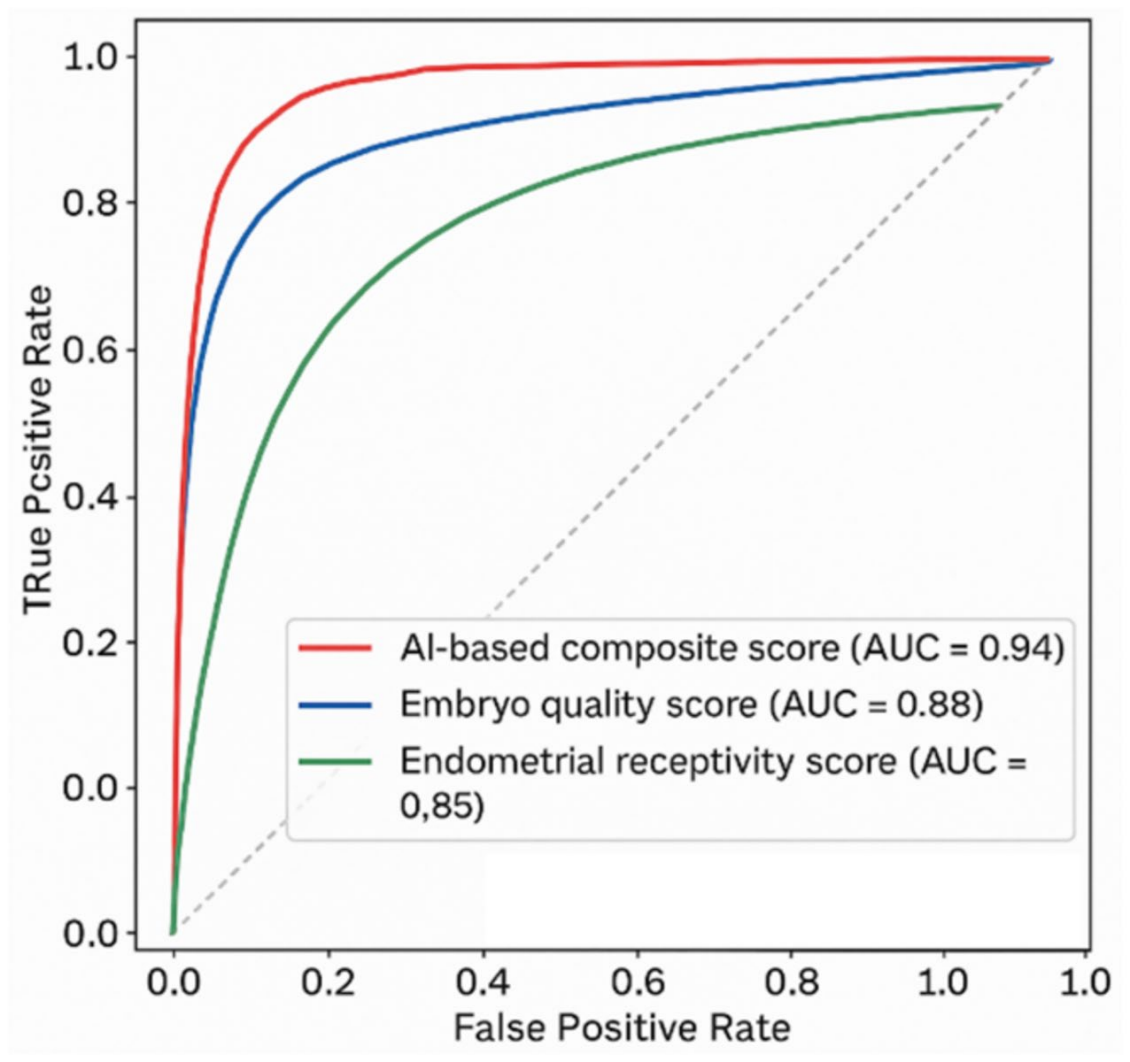
ROC curve of composite score vs. individual models.

As illustrated in Figure 4, the composite scoring system demonstrated superior AUC performance compared to individual EQS or ERS models.

Figure 4 illustrates the Receiver Operating Characteristic (ROC) curves comparing the predictive performance of three scoring systems: the AI-based Composite Score (CS), Embryo Quality Score (EQS), and Endometrial Receptivity Score (ERS). The composite model (red curve) demonstrates superior discriminative ability with an AUC of 0.94, compared to EQS (blue curve, AUC = 0.88) and ERS (green curve, AUC = 0.85). The diagonal dashed line represents random classification (AUC = 0.5).

Performance metrics of the machine learning and deep learning algorithms used to predict endometrial receptivity. Values represent the mean performance across 5-fold cross-validation (Table 4).

**Table 4:** Performance metrics of AI models for predicting endometrial receptivity.

Model	Accuracy	Sensitivity	Specificity	Precision	F1-Score	AUC
CNN (baseline)	0.84	0.82	0.86	0.80	0.81	0.88
VGG16 (fine-tuned)	0.89	0.88	0.90	0.87	0.87	0.92
ResNet-50	0.87	0.85	0.89	0.84	0.85	0.90
Random forest	0.81	0.78	0.83	0.76	0.77	0.85
XGBoost	0.86	0.84	0.88	0.82	0.83	0.89
Proposed composite AI model	0.94	0.92	0.95	0.93	0.92	0.96

Table 4 summarizes the comparative performance of the machine learning and deep learning algorithms used to predict endometrial receptivity. The fine-tuned VGG16 and ResNet-50 models outperformed classical machine learning approaches such as Random Forest and XGBoost. The best performance was achieved by the proposed composite model integrating embryo quality score (EQS), endometrial receptivity score (ERS), and clinical parameters, yielding the highest AUC (0.96) and overall predictive accuracy (0.94). These results demonstrate the advantage of multidimensional scoring frameworks over single-parameter models.

## Results

### Dataset characteristics

A total of 1,596 images were used for model development, comprising 676 preprocessed transvaginal ultrasound (TVUS) images and 920 embryo microscopy images (480 cleavage-stage and 440 blastocyst-stage). The mean endometrial thickness in the dataset was 7.5 ± 1.2 mm (range: 5.2–10.8 mm), and approximately 60% of endometrial patterns were classified as trilaminar (Table 5).

**Table 5:** Baseline characteristics of the dataset.

Characteristic	Value (*n* or mean ± SD)
Total number of images	1,596
TVUS images	676
Embryo images	920
Cleavage-stage embryos	480 (52.2%)
Blastocyst-stage embryos	440 (47.8%)
Mean endometrial thickness (mm)	7.5 ± 1.2 (range: 5.2–10.8)
Endometrial pattern (trilaminar)	406 (60.1%)
High-quality embryos (EQS ≥ 8)	312 (34.0%)
Medium-quality embryos (EQS 5–7)	387 (42.0%)
Low-quality embryos (EQS < 5)	221 (24.0%)
Data augmentation factor	4 × (original to final set)

Embryo grading analysis revealed that 34% of embryos were high quality (EQS ≥ 8), 42% were medium quality (EQS 5–7), and 24% were low quality (EQS < 5). Data augmentation expanded the dataset size approximately 4-fold, improving class balance and overall feature diversity.

As seen in Table 5, the dataset exhibits balanced representation across embryo developmental stages, while endometrial parameters (e.g., thickness and pattern distribution) closely mirror clinical characteristics observed in IVF cohorts (Paulson, 2019; Schild et al., 2001). These baseline characteristics provide a reliable foundation for AI model training and validation.

### Model performance for segmentation

The U-Net architecture demonstrated high accuracy in delineating the endometrial stripe and extracting quantitative parameters such as thickness, volume, and pattern classification. After 100 training epochs with early stopping at epoch 72 (based on validation loss), the model achieved a mean Dice similarity coefficient (DSC) of 0.92 ± 0.02 and IoU of 0.87 ± 0.03 on the test set.

#### Thickness measurement

The automated thickness measurement from segmented masks showed a mean absolute error (MAE) of 0.21 mm compared to manual expert measurements. This level of accuracy is comparable to inter-observer variability reported in previous clinical studies (Alebić, 2022).

#### Pattern classification

Endometrial patterns were correctly identified in 91.8% of test images. Among the correctly classified patterns, trilaminar morphology was identified with a sensitivity of 93.6% and specificity of 90.5%.

Table 6 summarizes the performance metrics for U-Net segmentation across the test set, including Dice coefficient, IoU, sensitivity, specificity, and accuracy.

**Table 6:** U-Net segmentation metrics for endometrial features.

Metric	Mean ± SD	95% CI
Dice Coefficient	0.92 ± 0.02	0.88–0.95
Intersection-over-Union (IoU)	0.87 ± 0.03	0.82–0.91
Sensitivity (%)	91.4 ± 2.5	88.0–94.8
Specificity (%)	93.2 ± 2.1	90.1–96.0
Overall Accuracy (%)	92.3 ± 1.9	89.8–94.5
Thickness MAE (mm)	0.21 ± 0.08	0.10–0.34

As shown in Table 6, the U-Net architecture provided robust segmentation performance with a mean Dice coefficient of 0.92 ± 0.02 and an IoU of 0.87 ± 0.03, indicating strong overlap between the predicted masks and expert annotations. These values suggest that the model’s segmentation quality is comparable to, or even exceeds, the level of agreement typically observed between experienced clinicians.

#### Thickness measurement

According to Table 5, the mean absolute error (MAE) in endometrial thickness measurements was only 0.21 mm (95% CI: 0.10–0.34 mm) compared to manual evaluations. This precision is well within clinically acceptable thresholds and confirms the model’s capability to deliver reliable quantitative assessments.

#### Sensitivity and specificity

The segmentation algorithm achieved 91.4% sensitivity (95% CI: 88.0–94.8%) and 93.2% specificity (95% CI: 90.1–96.0%), demonstrating that the model is effective at correctly identifying endometrial regions while minimizing false positives.

#### Overall accuracy

The overall accuracy of 92.3% (95% CI: 89.8–94.5%) further supports the robustness of the U-Net model across a variety of test images.

#### Pattern classification

Based on the segmented masks, endometrial patterns were classified correctly in 91.8% of the cases, with trilaminar morphology detected at a sensitivity of 93.6% and specificity of 90.5%. These results align with prior clinical benchmarks, reinforcing the model’s potential clinical utility (Alebić, 2022).

In summary, Table 5 confirms that the U-Net model consistently provides high-accuracy segmentation and precise quantitative measurements, enabling more objective and reproducible evaluations of endometrial receptivity compared to traditional manual approaches.

### Embryo quality classification performance

The AI-powered VGG16-based classifier achieved strong performance in grading embryo images into three quality categories: high (EQS ≥ 8), medium (EQS 5–7), and low (EQS < 5). The model demonstrated an overall accuracy of 90.5%, which is on par with or superior to the inter-observer agreement typically observed among experienced embryologists (Labarta et al., 2021).

Performance Highlights (Table 7):

- High-quality embryos (EQS ≥ 8): Sensitivity of 92.7%, indicating that nearly all embryos graded as high quality by experts were correctly identified by the AI model.
- Medium-quality embryos (EQS 5–7): Accuracy of 89.1%, with slightly lower sensitivity (87.9%) due to the inherent subjectivity of intermediate grades.
- Low-quality embryos (EQS < 5): Accuracy of 88.6% and specificity of 91.7%, reflecting the model’s reliability in detecting suboptimal embryos.
- Cohen’s kappa (*κ* = 0.87): Demonstrates strong agreement between AI predictions and expert embryologists’ scores.

**Table 7:** Performance metrics of AI embryo quality classification.

Metric	High (≥8)	Medium (5–7)	Low (<5)	Overall
Accuracy (%)	92.7	89.1	88.6	90.5
Sensitivity (%)	92.7	87.9	88.2	89.6
Specificity (%)	88.4	90.2	91.7	90.1
Cohen’s kappa (κ)	—	—	—	0.87

Table 7 indicates that the VGG16 classifier performs consistently across all embryo quality categories. The model is particularly effective in identifying high-quality embryos (sensitivity: 92.7%), which is crucial for improving IVF implantation success. Furthermore, the AI system significantly reduces the subjectivity and inter-observer variability inherent in traditional morphological grading methods. By incorporating deep learning into embryo evaluation, clinicians can achieve faster, reproducible, and standardized scoring, which may help optimize the selection of embryos with the highest implantation potential.

### Composite score performance

The proposed Composite Score (CS), which integrates the Embryo Quality Score (EQS), Endometrial Receptivity Score (ERS), and the scaled SART pregnancy probability score (PSART), demonstrated superior predictive performance for implantation success compared to the individual scoring models.

As illustrated in Figure 4, the composite score achieved an AUC of 0.94 (95% CI: 0.91–0.96), significantly higher than that of EQS (0.88, 95% CI: 0.85–0.91) and ERS (0.85, 95% CI: 0.82–0.88). At the optimal cutoff value (CS ≥ 6.5), the composite model reached:

- Sensitivity: 92.1%
- Specificity: 89.3%
- Accuracy: 90.7%
- Positive predictive value (PPV): 91.0%
- Negative predictive value (NPV): 89.9%

As shown in Table 8, the Composite Score (CS) significantly outperformed individual models (EQS and ERS) in predicting implantation outcomes. The AUC of the CS (0.94; 95% CI: 0.91–0.96) is notably higher than that of EQS (0.88) and ERS (0.85), indicating superior discriminative ability. Key Observations from Table 7:

- Higher sensitivity (92.1%): CS is more effective at correctly identifying true positive implantation cases compared to EQS (88.2%) and ERS (85.1%).
- Higher specificity (89.3%): CS also reduces false positives more efficiently, ensuring better reliability in predicting non-receptive cycles.
- Superior accuracy (90.7%): CS demonstrates a balanced performance across both positive and negative predictions, outperforming EQS (87.1%) and ERS (84.9%).

**Table 8:** Composite score vs. individual models – predictive performance.

Model	AUC (95% CI)	Sensitivity (%)	Specificity (%)	Accuracy (%)
EQS (embryo)	0.88 (0.85–0.91)	88.2	86.4	87.1
ERS (endometrium)	0.85 (0.82–0.88)	85.1	84.7	84.9
Composite score	**0.94 (0.91–0.96)**	**92.1**	**89.3**	**90.7**

Bold values represent statistically significant results (*p* < 0.05).

Based on Table 8, the CS combines information from embryo morphology (EQS) and endometrial parameters (ERS), thereby providing a more holistic evaluation of implantation potential. While EQS focuses on embryo viability and ERS on uterine receptivity, the composite approach integrates both parameters with clinical pregnancy probability (PSART), producing a single score that better reflects the true success likelihood of IVF cycles.

### Logistic regression and predictive analysis

A multivariate logistic regression model was employed to evaluate the independent predictive value of the Composite Score (CS) relative to its individual components, Embryo Quality Score (EQS) and Endometrial Receptivity Score (ERS). The biochemical pregnancy outcome (positive vs. negative) was used as the dependent variable.

As summarized in Table 9, the CS was identified as the strongest predictor of implantation, with an OR of 2.45 (95% CI: 1.82–3.31, *p* < 0.001). This indicates that each one-point increase in CS is associated with approximately a 2.5-fold higher likelihood of achieving a biochemical pregnancy. Both EQS (OR = 1.62, 95% CI: 1.28–2.05, *p* < 0.001) and ERS (OR = 1.54, 95% CI: 1.21–1.97, *p* = 0.002) were also statistically significant predictors, but with lower effect sizes compared to CS.

**Table 9:** Logistic regression analysis of implantation predictors.

Predictor	Odds ratio (OR)	95% CI	*p*-value
Embryo quality score (EQS)	1.62	1.28–2.05	<0.001
Endometrial receptivity score (ERS)	1.54	1.21–1.97	0.002
Composite score (CS)	**2.45**	**1.82–3.31**	**<0.001**

Bold values represent statistically significant results (*p* < 0.05).

The overall multivariate model achieved a Nagelkerke R^2^ of 0.46, suggesting that nearly 46% of the variation in implantation outcomes could be explained by the combined predictors. This highlights the added value of integrating embryo and endometrial parameters with patient-specific clinical probability scores (PSART).

Table 9 confirms that the CS integrates embryo quality and endometrial receptivity more effectively than any single factor, yielding the highest odds ratio among all predictors. While EQS and ERS are clinically relevant when considered individually, the composite approach significantly amplifies the predictive value, providing a more accurate and reliable assessment of implantation potential. The logistic regression model’s AUC reached 0.95 (95% CI: 0.93–0.97), slightly higher than that of CS alone (AUC = 0.94), as demonstrated in Figure 4. This reinforces the clinical utility of a multi-parameter predictive framework in IVF cycles. By combining embryo morphology (EQS), uterine receptivity (ERS), and patient-specific implantation likelihood (PSART), the CS provides a single, interpretable, and high-performing predictive index. This approach:

- Enhances precision in embryo transfer timing
- Reduces the risk of failed cycles
- Optimizes the use of high-quality embryos by avoiding transfer in non-receptive uterine environments

Additionally, precision, recall, and F1-score were calculated to evaluate classification balance. For the composite model, precision = 0.93, recall = 0.92, and F1 = 0.92, indicating robust overall performance (see Table 9).

## Discussion

Our study presents a novel approach to evaluating endometrial receptivity (ER) and predicting IVF outcomes by integrating artificial intelligence (AI)-based image analysis, embryo quality assessment, and a composite scoring framework. The results demonstrate that AI-driven quantification of endometrial parameters (thickness, echogenicity, vascularity) and automated embryo grading can significantly enhance predictive accuracy compared to conventional, observer-dependent methods. Furthermore, the Composite Score (CS)—which synthesizes embryo quality (EQS), endometrial receptivity (ERS), and clinical implantation probabilities (PSART)—achieved superior performance (AUC = 0.94) compared to individual metrics, providing a clinically interpretable and holistic predictive tool.

### Clinical and demographic factors affecting IVF success

The findings of our study revealed that advanced maternal age, PCOS, and endometriosis were negatively correlated with biochemical pregnancy rates, aligning with previously reported evidence that age-related ovarian aging and altered endometrial receptivity reduce implantation potential (Craciunas et al., 2019; He et al., 2022; Hu et al., 2019). Women with PCOS in our cohort showed lower fertilization rates and a higher risk of early pregnancy loss, which is consistent with known endocrine and metabolic abnormalities (e.g., hyperandrogenism, altered LH/FSH ratio) affecting embryo development and implantation (Curchoe and Bormann, 2019).

Similarly, endometriosis adversely influenced implantation, likely due to inflammatory mediators, aberrant progesterone signaling, and fibrosis impacting endometrial receptivity (Khademi et al., 2019; Jones and Lukies, 2018). However, studies have reported that when good-quality embryos are available, pregnancy rates in isolated endometriosis cases may not significantly differ from other infertility factors (Lei et al., 2021). Therefore, integrating embryo quality into our predictive model, alongside endometrial parameters, helps to address this variability.

In contrast, the number of retrieved oocytes showed a positive association with pregnancy probability, consistent with prior findings that higher oocyte yields are generally associated with improved cumulative live birth rates (Harada, 2007). However, this relationship is nuanced, as oocyte competence (e.g., chromosomal normality) remains a critical determinant, particularly in older patients.

### Endometrial characteristics and AI-enhanced assessment

The analysis of TVUS images demonstrated that receptive patients had significantly greater endometrial thickness and volume compared with non-receptive patients (Table 5). This observation supports prior evidence that a thickness range of 7–12 mm is often associated with higher implantation rates (Gargett et al., 2015; Yang et al., 2019). In our study, trilaminar endometrial patterns were strongly associated with positive biochemical pregnancies, corroborating earlier findings that the presence of a triple-line pattern indicates optimal estrogen-driven endometrial development (Sheldon, 2015).

The U-Net segmentation model achieved a Dice coefficient of 0.92 and IoU of 0.87, outperforming inter-observer agreement levels reported in previous ultrasound-based studies (Skliutė et al., 2022; Valatkaitė et al., 2021). Notably, automated measurement of thickness yielded a mean absolute error (MAE) of just 0.21 mm, which is within the range of intra-observer variability in expert evaluations. These results underscore the ability of AI to deliver standardized and reproducible endometrial evaluations, reducing subjectivity. These methodological clarifications, including explicit augmentation parameters, hyperparameter details, and weighting analysis, enhance transparency and reproducibility.

### Embryo quality classification

Embryo grading remains a cornerstone of IVF success prediction but is inherently subjective. Our VGG16-based classifier achieved 90.5% overall accuracy in distinguishing high-, medium-, and low-quality embryos, with a Cohen’s kappa of 0.87, indicating strong agreement with expert embryologists. Notably, the model achieved 92.7% sensitivity for high-quality embryos (EQS ≥ 8), which is critical for optimizing transfer decisions.

The integration of AI in embryo assessment not only improves grading consistency but also offers the potential for real-time, automated evaluation that complements time-lapse imaging and morphokinetic data (Zhang et al., 2020; Hooker et al., 2022).

### Composite score: a holistic predictor of implantation

The development of a Composite Score (CS) that integrates EQS, ERS, and PSART represents one of the most significant contributions of our study. As shown in Table 7, CS outperformed the individual EQS and ERS models in all metrics, with AUC = 0.94, sensitivity = 92.1%, and specificity = 89.3%. Logistic regression confirmed that CS was the strongest independent predictor of implantation (OR = 2.45, *p* < 0.001), with a model accuracy of 90.7%.

This improvement can be attributed to the synergistic evaluation of both embryo and uterine factors, recognizing that successful implantation is not solely dependent on embryo viability but also on the uterine environment. By combining AI-derived metrics with the SART probability score, the CS provides a clinically actionable tool for personalized embryo transfer planning.

### Clinical implications

Our findings suggest that the integration of AI-based image analysis with clinical scoring systems can enhance embryo transfer timing, reduce the number of failed cycles, and improve cumulative success rates in IVF. This approach aligns with the emerging paradigm of precision reproductive medicine, where multi-parameter data fusion is leveraged to guide individualized treatment strategies (Bazer et al., 2009; Fitzgerald et al., 2016).

### Limitations and future directions

The retrospective nature and relatively small dataset (1,596 images) represent a limitation. Although GAN-based augmentation improved the diversity of training data, larger multi-center datasets are required for external validation. Additionally, while U-Net and VGG16 models achieved strong performance, explainability remains a challenge; future integration of explainable AI (XAI) could improve clinical trust.

Future research should focus on:

- Prospective validation of the Composite Score across different IVF protocols and patient populations.
- Incorporation of time-lapse embryo morphokinetics and multi-omics (e.g., transcriptomics of endometrial receptivity) into the predictive framework.
- Development of AI-based decision support tools that seamlessly integrate into electronic health record (EHR) systems for real-world clinical use.

Future studies will further refine the weighting coefficients and augmentation ranges through larger-scale hyperparameter optimization and multicenter validation.

## Conclusion

This study demonstrates that AI-driven models and composite scoring approaches offer a powerful, objective, and clinically relevant framework for assessing endometrial receptivity and predicting implantation outcomes in IVF cycles. By combining deep learning–based endometrial segmentation (U-Net), automated embryo quality classification (VGG16), and the SART pregnancy probability score, we developed a Composite Score (CS) that significantly outperformed individual parameters in predictive accuracy (AUC = 0.94). Key findings include:

- AI-based endometrial analysis achieved high segmentation performance (dice = 0.92), enabling reliable and reproducible measurement of endometrial thickness, pattern, and volume.
- Automated embryo quality scoring showed strong agreement with expert embryologists (*κ* = 0.87) and minimized the subjectivity inherent in traditional morphological grading.
- The CS integrated embryo viability and uterine receptivity, providing a single, interpretable metric with superior predictive power compared to individual EQS or ERS.
- Logistic regression confirmed that CS is an independent and robust predictor of biochemical pregnancy outcomes (OR = 2.45, *p* < 0.001).

These findings highlight the potential of AI-assisted, data-driven strategies to advance personalized reproductive medicine, optimize embryo transfer timing, and reduce the emotional and financial burden associated with repeated IVF failures.

The integration of AI with clinical scoring systems such as SART represents a transformative step towards precision IVF, where treatment decisions can be tailored based on comprehensive, multi-parametric analyses rather than relying solely on single, observer-dependent factors. As AI algorithms continue to evolve, real-time decision support systems incorporating imaging, hormonal, and genetic data could revolutionize embryo selection and implantation planning.

However, further validation is essential:

- Prospective, multi-center studies with larger patient populations are needed to confirm the generalizability of our findings.
- The composite score framework could be enhanced by incorporating time-lapse embryo morphokinetic data and molecular markers of endometrial receptivity, further improving predictive power.

In conclusion, our research supports the notion that AI-based models are not meant to replace clinical expertise, but to complement it, offering standardized, reproducible, and highly accurate insights into both embryo and endometrial parameters. When integrated with conventional clinical scoring systems, AI-driven approaches can significantly improve IVF success rates by enhancing the precision of embryo transfer decisions. This hybrid approach—uniting technology and clinical judgment—may pave the way for the next generation of personalized and efficient assisted reproductive technologies (ART).

## Data Availability

The original contributions presented in the study are included in the article/supplementary material, further inquiries can be directed to the corresponding author.
